# In cultured cells the baculovirus P10 protein forms two independent intracellular structures that play separate roles in occlusion body maturation and their release by nuclear disintegration

**DOI:** 10.1371/journal.ppat.1007827

**Published:** 2019-06-10

**Authors:** Leo P. Graves, Louise C. Hughes, Sarah L. Irons, Robert D. Possee, Linda A. King

**Affiliations:** 1 Department of Biological and Medical Sciences, Oxford Brookes University, Headington, Oxford, United Kingdom; 2 Oxford Expression Technologies Ltd, Oxford, United Kingdom; Florida State University, UNITED STATES

## Abstract

P10 is a small, abundant baculovirus protein that accumulates to high levels in the very late stages of the infection cycle. It is associated with a number of intracellular structures and implicated in diverse processes from occlusion body maturation to nuclear stability and lysis. However, studies have also shown that it is non-essential for virus replication, at least in cell culture. Here, we describe the use of serial block-face scanning electron microscopy to achieve high-resolution 3D characterisation of P10 structures within *Trichoplusia ni* TN-368 cells infected with Autographa californica multiple nucleopolyhedrovirus. This has enabled unparalleled visualisation of P10 and determined the independent formation of dynamic perinuclear and nuclear vermiform fibrous structures. Our 3D data confirm the sequence of ultrastructural changes that create a perinuclear cage from thin angular fibrils within the cytoplasm. Over the course of infection in cultured cells, the cage remodels to form a large polarised P10 mass and we suggest that these changes are critical for nuclear lysis to release occlusion bodies. In contrast, nuclear P10 forms a discrete vermiform structure that was observed in close spatial association with both electron dense spacers and occlusion bodies; supporting a previously suggested role for P10 and electron dense spacers in the maturation of occlusion bodies. We also demonstrate that P10 hyper-expression is critical for function. Decreasing levels of *p10* expression, achieved by manipulation of promoter length, correlated with reduced P10 production, a lack of formation of P10 structures and a concomitant decrease in nuclear lysis.

## Author summary

High-resolution 3D electron microscopy has revealed the complexity of structures formed by P10, a small 10kDa protein that accumulates to very high levels in baculovirus-infected cells. We demonstrate the formation and presence of two distinct, possibly unique, P10 structures that account for the diverse roles associated with this small protein. In the cytoplasm, a peri-nuclear cage-like structure matured into a polarised mass of P10. Remodelling of the cage provides evidence for a mechanism to effect nuclear lysis and release of occlusion bodies to promote dispersal. Over a similar time period, an independent vermiform P10 structure forms and matures within the cell nucleus. It is widely known that in the absence of P10, occlusion bodies do not fully mature. Our data suggest a mechanism for occlusion body maturation with P10 facilitating the envelopment of occlusion bodies with electron dense spacers. The P10 structures formed require vast quantities of P10 protein providing a rationale for the hyper-expression of this hitherto obscure viral protein.

## Introduction

The Baculoviridae is a diverse family of insect-specific viruses that contain circular, supercoiled dsDNA genomes ranging from 80 to 180 kilobase pairs (kbp) [1, 2]. Baculoviruses are characterised by rod shaped virions between 250–400 nm in length and 30–70 nm in diameter [3, 4] that are embedded in protective paracrystalline protein structures known as polyhedra or occlusion bodies (OBs) [5]. Protection of virus particles within OBs is primarily restricted to invertebrate-specific viruses and is thought to relate to the need to preserve virus during diapause or when host larval numbers are scarce [6–9]. The nature of baculoviruses has led to their widespread use in agriculture, as a bio-pesticide [6] and as a platform for gene expression and recombinant protein production [10, 11]. The expression system has exploited the highly efficient promoters of the *p10* and polyhedrin (*polh*) genes, both of which have been found to be non-essential for the propagation of the virus in cell culture [12–14].

Over the course of a baculovirus infection, whether in larval tissue or in cultured insect cells, cell nuclei become enlarged and, in the very-late stages, become packed with the para-crystalline OBs that are readily visible under the light microscope [15]. Ultimately, nuclei disintegrate releasing OBs or polyhedra into the liquefied larval remains or cell culture medium [16, 17]. The major component of OBs is the 30 kDa polyhedrin protein that is produced under control of the very late, hyper-expressed *polh* promoter [18]. During the final stage of maturation, OBs acquire a polyhedral envelope (PE)/calyx, which largely consists of a polyhedral envelope protein (PEP), PP34 in Autographa californica multiple nucleopolyhedrovirus (AcMNPV) [19–21], and a carbohydrate matrix. The process for PE formation is still the subject of speculation. However, both P10 and electron dense spacers (EDS) associate with or contain pp34 (39–40) and appear to play a key role in PE formation [5, 15, 22, 23]. In particular, it has been shown that in the absence of P10 or pp34, OBs do not fully mature leaving a rough and pitted surface [22].

The abundant P10 protein is produced from the other very-late, hyper-expressed gene found in the baculovirus genome [10]. Early observations of baculovirus-infected cells by transmission electron microscopy (TEM) noted the formation of large fibrous structures in both the nucleus and cytoplasm during the later phases of infection [5, 23–25]. Initially it was proposed that these fibrous structures represented the polyhedrin protein in its physical state prior to formation of OBs; largely due to the close association with developing OBs. It was not until later, with the characterisation of polyhedrin and a 10-kDa protein, referred to as P10 [10, 11, 26–28], that these fibrous structures were identified as separate to OBs [29].

Evidence from TEM and confocal microscopy suggest P10 is associated with a number of intracellular structures with potential roles not only in OB maturation [15, 19, 22], but also nuclear stability [30] and mediation of nuclear disintegration [17]. Immunofluorescence studies on AcMNPV-infected *Trichoplusia ni* (TN-368) or *Spodoptera frugiperda* (Sf9) cells, using anti-tubulin and anti-P10 antibodies, observed that the initial thinner P10 structures co-localised with microtubules [27, 28]. These studies suggested that cross linking with microtubules may promote the reorganisation of the cytoskeleton that is observed in baculovirus-infected cells and may be a prerequisite for P10 self-assembly [30, 31]. Interestingly, however, the thicker P10 structures found from 48 hpi do not co-localise with microtubules [30]. Instead, confocal microscopy indicated that they form a perinuclear network or ‘cage’ enveloping the infected cell nucleus [28]. To date, the mechanisms underlying the formation and role of these structures is unknown.

Most of our knowledge of the structures associated with P10 has come from studies using confocal microscopy [28]. Understanding of the detailed ultrastructure may help elucidate how and why these structures form. To enable visualisation of the three dimensional (3D) ultrastructure, we chose to use serial block-face scanning electron microscopy (SBFSEM) due to the combination of resolution that can be achieved and total cellular volume that can be imaged relatively rapidly. SBFSEM is an emerging technique that is rapidly gaining wider applications in many areas of biology [32, 33].

We report here the first use of SBFSEM to study baculovirus structures in infected insect cells. The remarkably detailed images show the formation of two independent and distinct P10 structures, one within the nucleus and one within the cytoplasm that is perinuclear. Additionally, our study has enabled the reconstruction of OBs and EDS and their close association with the nuclear form of P10.

The motivation for this study was to elucidate the functional roles of P10 during AcMNPV infection of cultured insect cells and to understand the mechanisms by which it can influence diverse processes. This includes events such as the maturation of OBs and nuclear disintegration, often referred to as nuclear lysis, for the release of mature OBs from the assembly location (nucleus) in to the cell culture medium (*in vitro*) or haemocoel (*in vivo*). Our results enable us to propose a unique model for the structural reorganisation of P10 over the course of the virus infection cycle that explains the multiple roles attributed to this small, abundant viral protein.

## Results

### P10 forms two independent structures during infection

**Fig 1:**
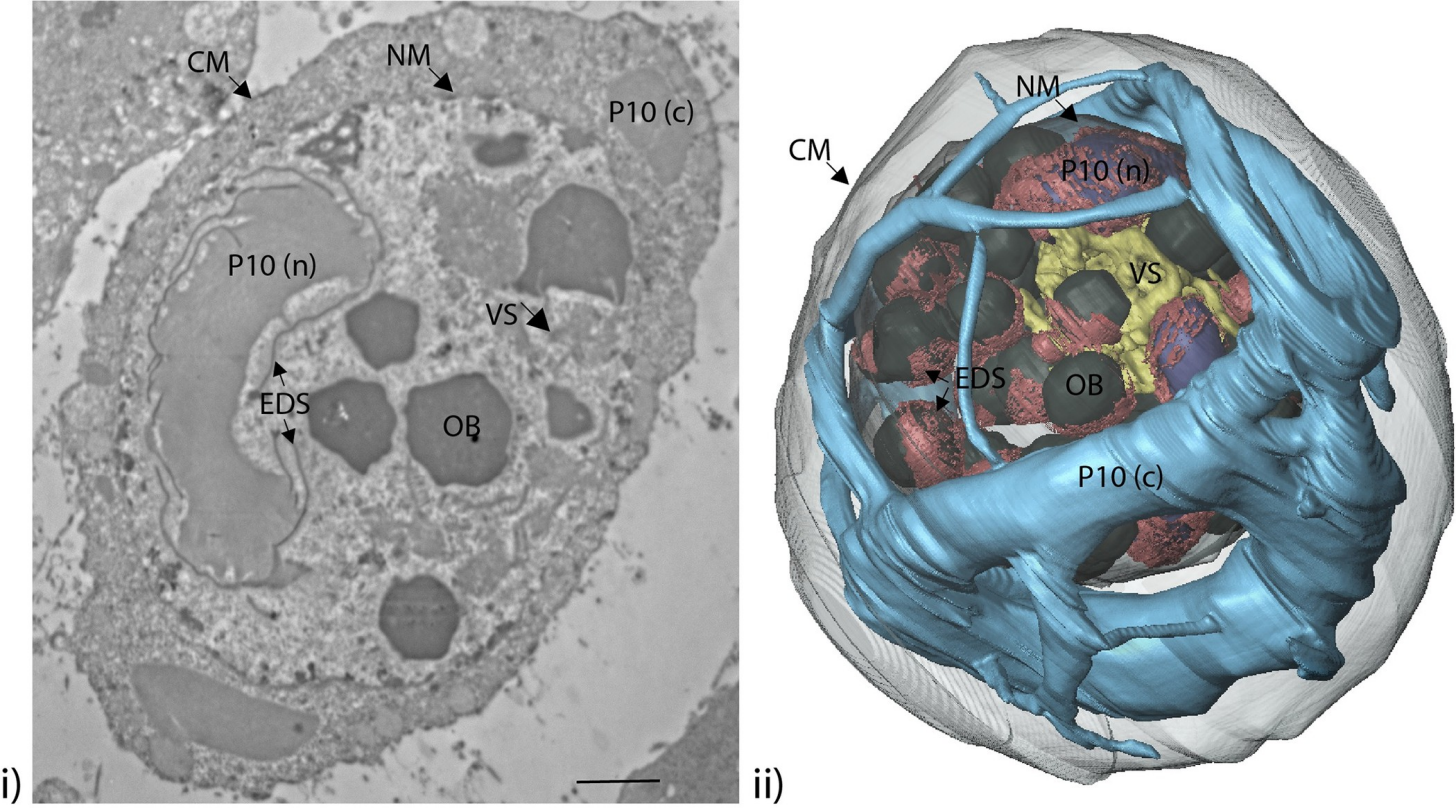
TEM image and SBFSEM reconstruction of baculovirus-infected cell structures. (i) TEM image of a typical AcMNPV-infected TN-368 cell at 72 hpi highlights typical virus-infected cell structures (arrowed): virogenic stroma (VS); P10 (nuclear, n; cytoplasmic, c); electron dense spacers (EDS); and occlusion bodies (OB) as well as the cytoplasmic (CM) and nuclear membranes (NM). (ii) Whole cell imaging using SBFSEM on a typical AcMNPV-infected TN-368 cell at 72 hpi has been used to generate a 3D reconstruction of the structures shown in the TEM image.

*Trichoplusia ni* (TN-368) cells were infected with AcMNPV at a multiplicity of infection (MOI) of 5 plaque forming units (pfu)/cell, and processed by embedding in resin for SBFSEM imaging at 24, 48, 72 and 96 hours post-infection (hpi). Image capture was optimised and typical baculovirus-infected cell structures such as the virogenic stroma (VS), P10, electron dense spacers (EDS) and occlusion bodies (OBs) were observed (Fig 1).

SBFSEM data were collected by imaging the cut surface of the resin-embedded AcMNPV-infected cells using a Gatan 3View system (Gatan, UK) in combination with a Zeiss Merlin compact VP scanning electron microscope (SEM, Zeiss, UK). A backscattered electron signal was used, which provided atomic contrast of the stained cells that was comparable to data collected using a TEM (Fig 2Ai). A diamond knife mounted within the specimen chamber was set to automatically slice the sample, with scanned images of the block face being captured between each 100nm slice [34, 35]. The subsequent stack of images (500–700) were aligned and segmented to produce 3D models of cellular and virus structures at different stages in the viral replication cycle as represented by 24, 48, 72 and 96 hpi (Fig 2Aii and 2Aiii; S1–S5 Videos).

**Fig 2:**
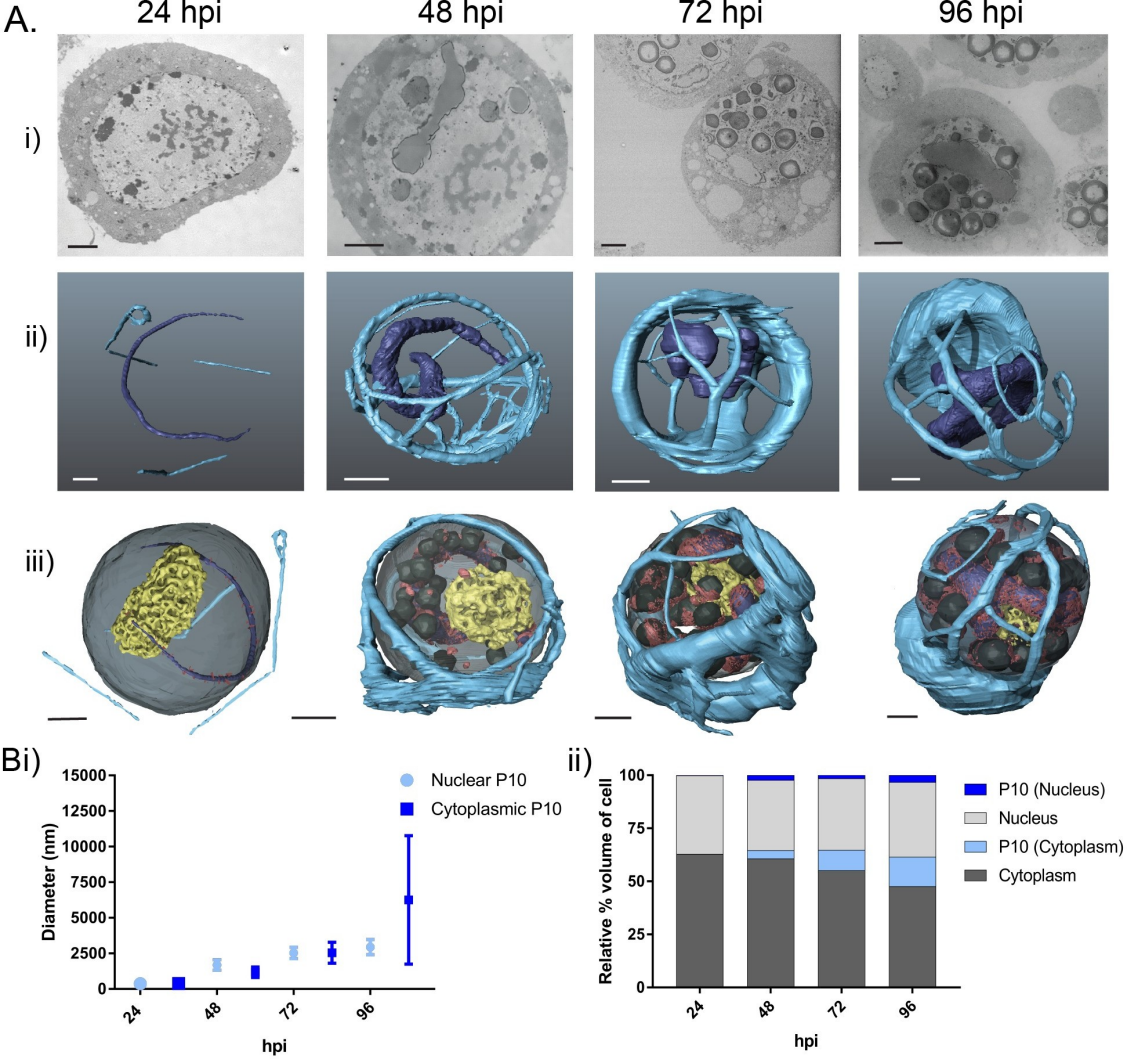
P10 structures in AcMNPV-infected TN-368 cells from 24 to 96 hours post-infection. (Ai) SEM images of resin embedded AcMNPV-infected TN-368 cells from the sample block face using the Gatan 3View system and a Zeiss Merlin compact field emission gun SEM at 24, 48, 72 and 96 hpi (see S2 Table for imaging parameters). (Aii) Whole cell 3D reconstruction (AMIRA) of nuclear and cytoplasmic P10 structures: nuclear P10 (dark blue), cytoplasmic P10 (light blue). (Aiii) P10 structures as in (Aii), plus reconstructed representations of virogenic stroma (yellow), occlusion bodies (dark grey) and electron dense spacers (red). (B) Estimate of P10 structure size using volumetric modelling data. (Bi) Datasets showing size range, mean measurements (nm) and (Bii) relative percentage volume of P10 structures compared to total cell (n = 2).

The P10 structures within AcMNPV-infected cells [36] were modelled at each time-point. Two distinct P10 fibrous structures were observed; a cytoplasmic perinuclear cage and a nuclear vermiform mass (Fig 2Aii). At 24 hpi, cytoplasmic P10 (light blue) was observed as thin filamentous branching structures. In contrast, at the same time point, nuclear P10 (dark blue) was observed as a single thin worm-like or vermiform structure (Fig 2Aii, 24 hpi). The two structures were separated by the host cell nuclear membrane (Fig 2Aiii, dark grey). At 48 hpi, the cytoplasmic P10 was demonstrably thicker than at 24 hours and had formed an elaborate network that enwrapped the nucleus (Fig 2Aii and 2Aiii, 48 hpi). At this time point, nuclear P10 had also increased in size, forming a thick elongated ‘worm’ (Fig 2Aii, 48 hpi). By 72 hpi, the cytoplasmic perinuclear P10 structure had remodelled to a form with fewer but thicker tubules (Fig 2Aii and 2Aiii, 72 hpi). However, at 72 hpi nuclear P10 (Fig 2Aii and 2Aiii, 72 hpi) appeared similar in morphology to that observed at 48 hpi.

By 96 hpi, a significant change in the morphology of the cytoplasmic P10 was apparent (Fig 2Aiii). The perinuclear cage structure was still present, but it lacked the extensive network of thick tubules observed at 48 and 72 hpi (Fig 2Aii and 2Aiii, 96 hpi). At this very late stage of the infection cycle, most P10 had coalesced to form a large, polarised aggregate, with a few remaining thin fibrils branching from it (Fig 2Aii and 2Aiii, 96 hpi). Simultaneously nuclear P10 maintained its characteristic long vermiform architecture (Fig 2Aii and 2Aiii, 96 hpi) and at this very late stage was surrounded by virogenic stroma and densely packed OBs.

### P10 structures increase in size between 24 and 96 hours post-infection

To investigate the changes in P10 over time, we estimated the diameter (nm) and relative cell volume (%) of the P10 structures. Representative diameter measurements were acquired manually in AMIRA using 3D volume rendered images of cytoplasmic P10 fibrils/tubules (n = 10) and nuclear P10 (n = 10) structures. These measurements were acquired from duplicate AcMNPV-infected cells modelled at 24, 48, 72 and 96 hpi (Fig 2Bi). AMIRA-generated 3D models of AcMNPV-infected cells were also used to calculate volumes of the cell nucleus, cytoplasm, nuclear P10 and cytoplasmic P10 to give an accurate representation of the changes that occurred during the virus replication cycle. In this study, the volumetric measurements are given as a percentage of the total cell volume (Fig 2Bii).

The combination of diameter and volumetric measurements from 24 to 96 hpi confirmed that both cytoplasmic and nuclear P10 structures increased in size as the infection cycle progressed. At 24 hpi, cytoplasmic and nuclear P10 structures had a mean diameter of 380 (± 186) nm and 370 (± 44) nm, respectively and together accounted for just 0.1% of the total cell volume (Fig 2Bi and 2Bii). By 48 hpi, the mean diameter of cytoplasmic P10 fibrils had nearly tripled in size to 1180 (± 367) nm, with nuclear P10 structures also increasing to 1680 (± 370) nm (Fig 2Bi). Volumetric measurements indicated a combined relative percentage increase to 6% of total cell volume. Cytoplasmic P10 structures continued to increase in diameter through 72 hpi but at this stage also demonstrated a wide range of diameters from 980 to 5520 nm (mean of 2547 ± 735 nm) with nuclear P10 recording a mean diameter of 2520 nm (Fig 2Bi). Volumetric readings confirmed these data, and at 72 hpi the P10 structures comprised 11% of the total cell volume (Fig 2Bii).

Measurements of the polarised aggregate of cytoplasmic P10 at 96 hpi correlated to the features observed both in 2D (Fig 2Ai) and 3D (Fig 2Aii and 2Aiii) microscopy, with the structure measuring as much as 14,150 nm diameter (mean 6258 ± 4511 nm) with the thin branching filaments at 520 nm. At this stage the diameter of nuclear P10 structures had increased to 2940 (± 530) nm (Fig 2Bi) and total P10 structures accounted for 17% of the relative percentage volume of the infected cell (Fig 2Bii).

### The 3D models constructed using SBF-SEM data are consistent with structures observed using confocal microscopy

**Fig 3:**
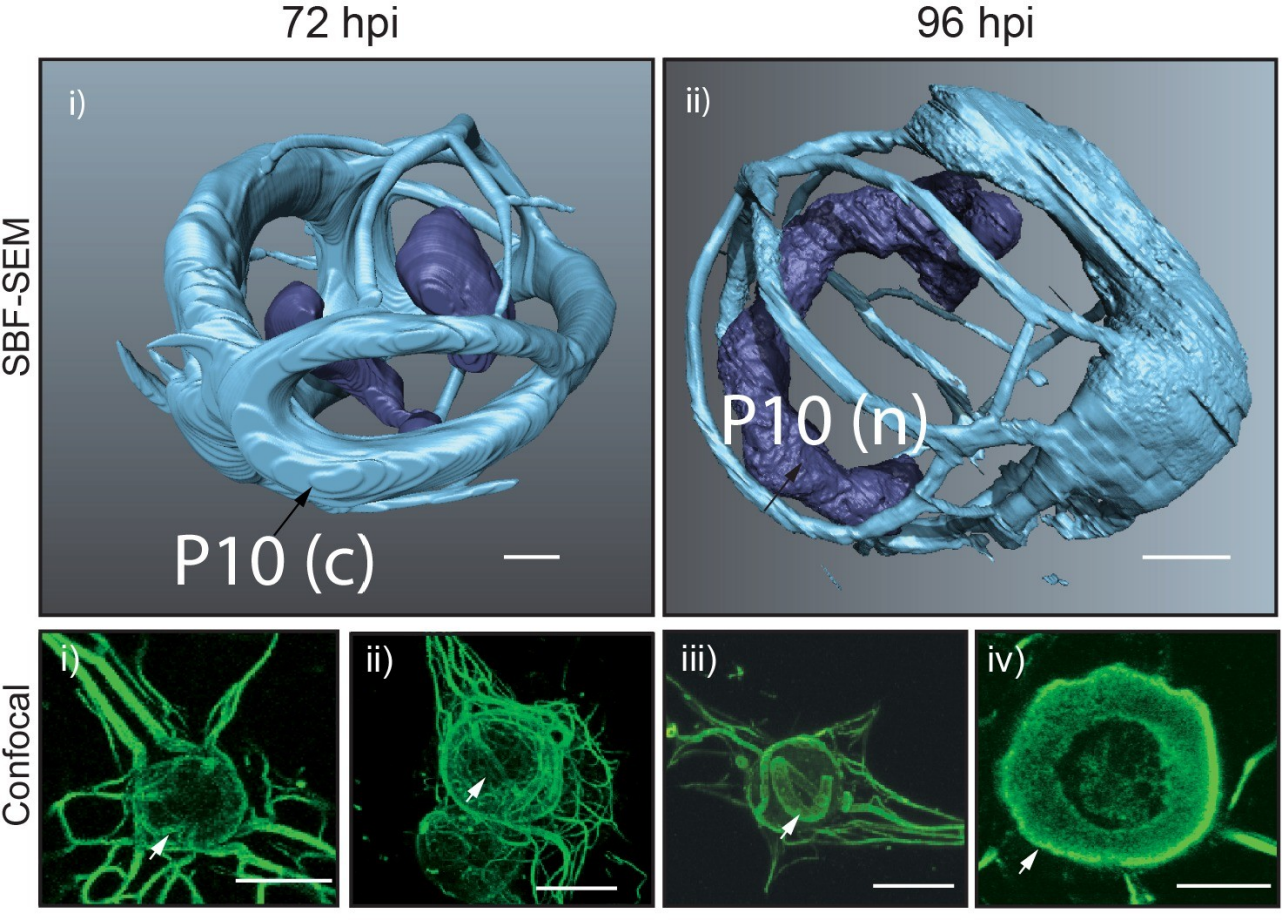
Surface generated images of AcMNPV-infected TN-368 cells at 72 hpi (i) and 96 hpi (ii) from SBF-SEM data sets. Corresponding confocal microscopy images of AcMNPV-infected TN-368 cells at 72 hpi (i–ii) and 96 hpi (iii-iv). Black arrows (SBFSEM) and white arrows (confocal) highlight P10 (green) features. Scale bar 3 μm (SBF-SEM) and 20 μm (confocal). Infected cells were fixed at 96 hpi and stained by indirect immunofluorescence using an anti-P10 antibody. A secondary antibody conjugated to an Alexa-fluor 488 was used to visualise P10 structures.

Analysis of AcMNPV-infected TN-368 cells using confocal microscopy showed the formation of P10 associated with thick peri-nuclear structures that encapsulated the nucleus at 72 hpi (Fig 3, Confocal, i and ii, arrow). During the later phase of infection at 96 hpi, P10 was associated with a thick cage-like structure around the nucleus and observed as a thick band (Fig 3, Confocal, iii and iv, arrow). The thinner cytoplasmic filaments had condensed and disassociated from the periphery of the nuclear membrane. This is in agreement with previously documented results [30]. These morphological structures observed using confocal microscopy are consistent with the 3D reconstruction of P10 using SBF-SEM (Fig 3, SBF-SEM).

### Modulation of P10 impacts nuclear disintegration

**Fig 4:**
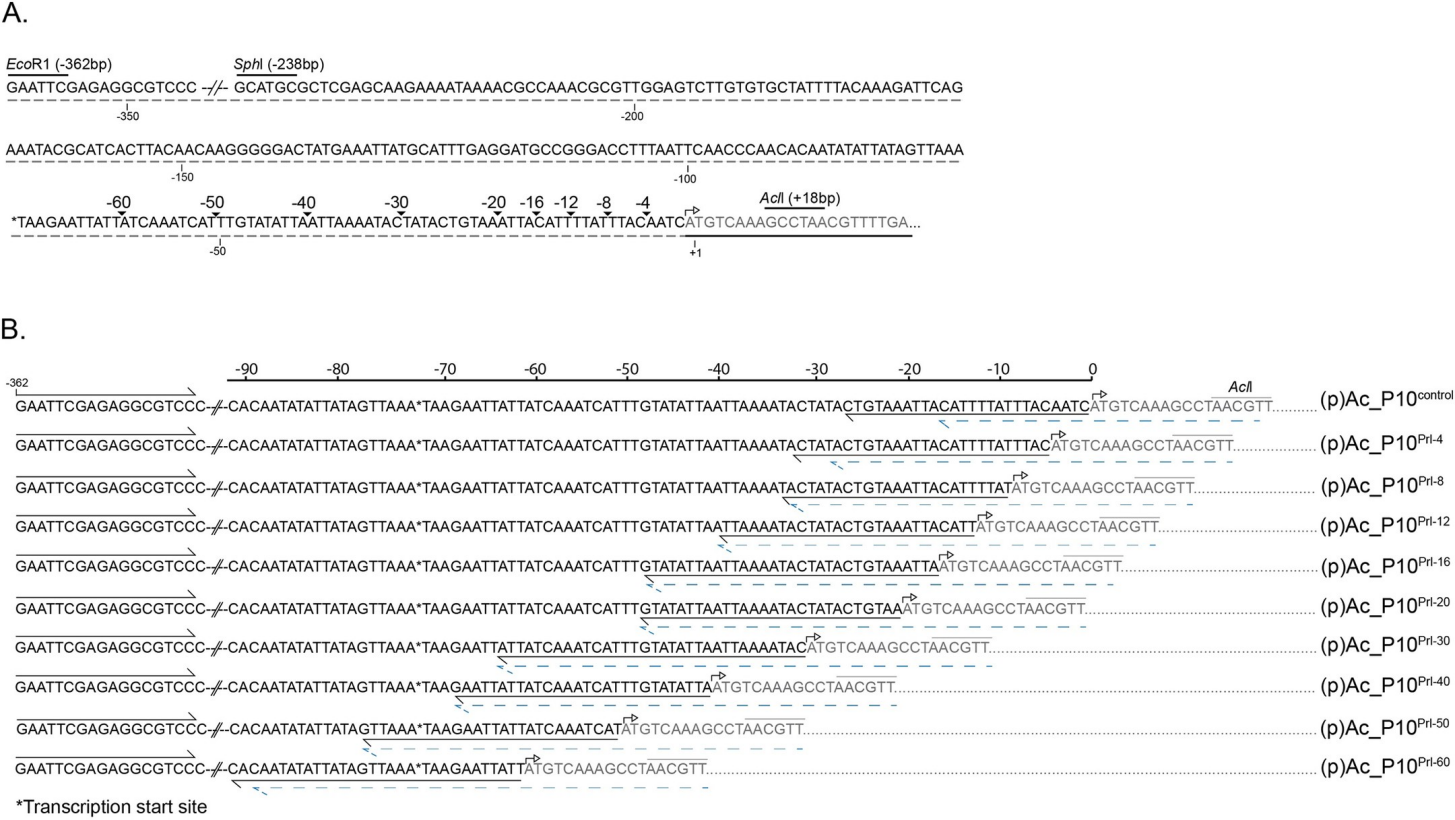
Nucleotide sequence of *p10* promoter leader sequence. (A) Indicates site for a series of deletions made to the promoter sequence from *transcription initiation (mRNA CAP) and location of relevant restriction digestion sites. (B) Displays nucleotide sequence of PCR fragments made using a two-step PCR to generate a series of promoter deletions as indicated in (A). The black arrows indicate binding of primer set for the first round PCR, the dotted blue line was use for the second round PCR and was used in combination with the forward primer upstream of the *p10* transcription start. The *p10* coding region starts at the ATG start codon as indicated by the open arrow and *p10* coding region is highlighted as grey text and underlined with a black line in (A).

**Fig 5:**
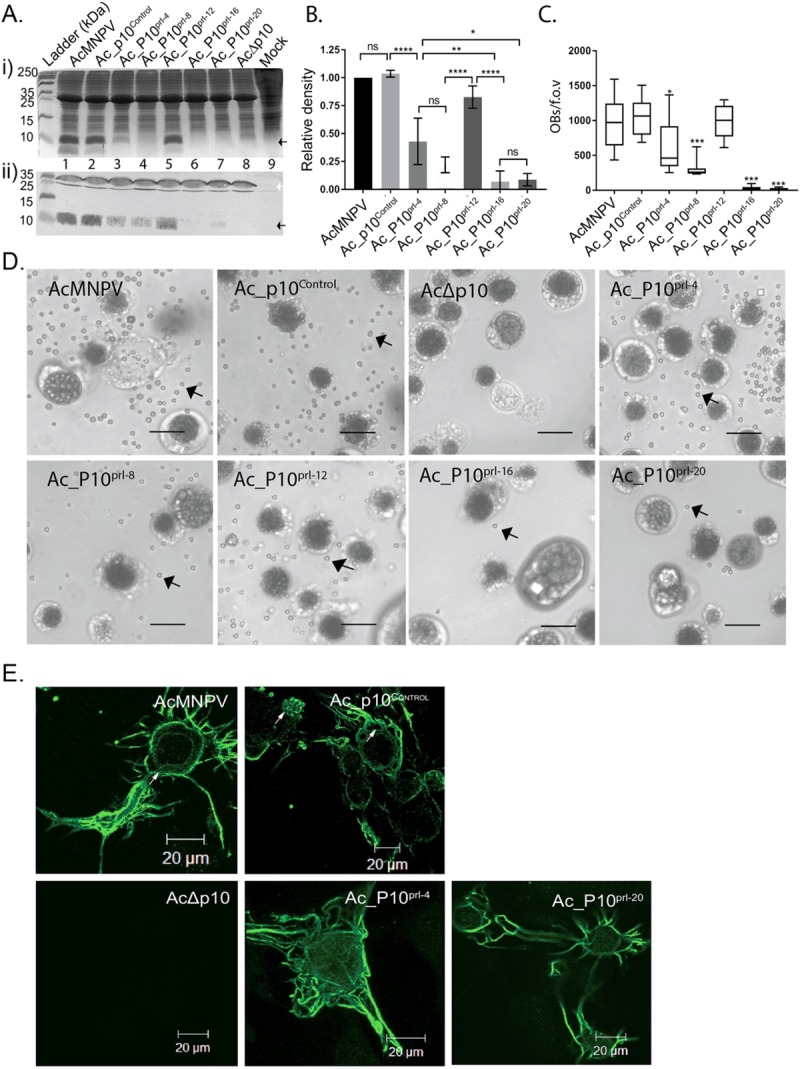
Effect of modulation of *p10* expression on P10 structure formation. (A) Infected TN-368 cells were harvested at 96 hpi and total protein fractionated in a 15% (w/v) SDS-PAGE for either Coomassie staining (i) or western blot analysis (ii) with P10- or cathepsin-specific antibodies detected using an alkaline phosphatase-conjugated secondary antibody. Lanes 1–9 (Aii) correlate to sample names at top (Aii). Arrows show expected sizes of P10 (black) and cathepsin (white). Ladder size (kDa) is indicated, left. (B) Bar graph showing mean relative P10 band density for AcMNPV, Ac_P10^Rescue^ Ac_P10^prl-4^, Ac_P10^prl-8^, Ac_P10^prl-12^, Ac_P10^prl-16^ and Ac_P10^prl-20^ from a series of western blots (n = 3) using Image J. (C) Box and whisker plot showing enumeration of released occlusion bodies of TN-368 cells infected with AcMNPV or recombinant viruses (5 MOI) at 7 dpi (n = 10 f.o.v images per virus). (D) TN-368 cells were infected with AcMNPV or recombinant viruses (5 MOI) in 35 mm dishes and incubated at 28°C. The cells were imaged using a light microscope Zeiss Axiovert 135 at 7 dpi to show cell morphology and lysis (100X). Scale bar is 20 μm. (E) Confocal microscopy of TN-368 cells infected with Ac_P10^Rescue^, Ac_P10^prl-4^ and Ac_P10^prl-20^ to observe P10 structures at 96hpi (green). Scale bar 20μm. Abbreviation: c cytoplasm, n nucleus, f.o.v field of view.

To test the hypothesis that hyper-expression of *p10* is critical for nuclear lysis, we constructed a series of AcMNPV *p10* promoter deletions within recombinant viruses (Fig 4). Controls for this experiment included recombinant viruses that either contained the full length *p10* promoter with the native P10 coding sequence (Ac_P10^Rescue^) or lacked the coding sequence (AcΔ*p10)*. After infection of cells with the control viruses or viruses containing promoter deletions, we showed using Coomassie staining (Fig 5Ai) and western blot analysis (Fig 5Aii) that there was a progressive decrease in P10 accumulation as base pairs within the *p10* promoter sequence were deleted (Fig 5Ai and 5Aii, lanes 3–7). The viruses used in this study contained 4, 8, 12, 16 or 20 base pair deletions in the *p10* 5’ non coding leader sequence beginning at the -1 position relative to the ATG.

Viruses designated Ac_P10^prl-16^ and Ac_P10^prl-20^ yielded levels of P10 that were below the detection limits for Coomassie staining. Interestingly, Ac_P10^prl-12^ displayed higher expression levels of P10 than that expected from neighbouring deletions, with a band density more comparable to AcMNPV and Ac_P10^Rescue^ (Fig 5Ai and 5Aii, lane 5). AcMNPV cathepsin was used as a marker to ensure equal loading on samples for the Western blot (Fig 5Aii). As expected, a P10-associated band was not detected for AcΔ*p10* either by Coomassie staining or Western blot (Fig 5, lane 8). By comparing band density on replicate Western blots (n = 3), a reduction in P10 expression with progressive removal of nucleotides from the *p10* promoter was confirmed (Fig 5B).

A number of functional roles have been suggested for P10 [reviewed in 37] as described above. Most distinctive in cell culture is the role of P10 in nuclear lysis or disintegration to facilitate dispersal of OBs [15, 17]. In the absence of P10, nuclear lysis does not occur and OBs are not released into the culture medium [15]. To determine whether hyper-expression of *p10* is required for nuclear lysis and release of OBs, *T*.*ni* Hi5 cells were infected with wild-type AcMNPV, Ac_P10^Rescue^, AcΔ*p10*, Ac_P10^prl-4^, Ac_P10^prl-8^, Ac_P10^prl-12^, Ac_P10^prl-16^ and Ac_P10^prl-20^ at 5 MOI and imaged at 7 days (d)pi using a light microscope (Fig 5D). A visual observation of virus-infected cells at 7 dpi indicated that the number of OBs released was directly related to the level of P10 synthesis (Fig 5B–5D). To confirm these results, the number of free OBs observed in a field of view at 7 dpi were enumerated using a haemocytometer (n = 10 for each virus) at 100X magnification (Fig 5C). The quantitative data confirmed the visual inspection results that Tni Hi5 cells infected with recombinant viruses lacking progressive deletions in the *p10* generated decreasing numbers of free OBs in the media (Fig 5C; P<0.05; one-way ANOVA). However, Ac_P10^prl-12^ had similar numbers of free OBs as counted for both AcMNPV (P>0.9999) and Ac_P10^Rescue^ (P>0.9918). No signs of nuclear lysis or free OBs were observed in AcΔ*p10-*infected cells (Fig 5C and 5D). This confirmed a relationship between P10 accumulation (Fig 5A nd 5B) and OB liberation (Fig 5C and 5D) and suggests modulation of P10 levels has a direct effect on nuclear lysis.

### The spatial and temporal association of P10 structures during infection indicate a role in nuclear disintegration

To further investigate the relationship between levels of P10 and nuclear lysis to release OBs, we infected TN-368 cells with the promoter deletion viruses Ac_P10^prl-4^ and Ac_P10^prl-20^ and examined the cells using confocal microscopy to observe any effects on P10 structure formation. We observed that reduced *p10* expression from the promoter deletion viruses (Fig 5A and 5B) impacted formation of P10 structures (Fig 5E). Reduced levels of expression was correlated with a delay in, or absence of, the formation of the thicker perinuclear tubular structures that form the cage-like structures characteristic of P10 in the very late stages of infection [30]. The control wild-type AcMNPV and Ac_P10^Rescue^ virus formed a P10-associated cage-like structure around the nucleus as expected. In Ac_P10^prl-4^-infected TN-368 cells, P10 was observed only as thinner, peri-nuclear cytoplasmic filaments with some branching out in the cytoplasm (Fig 5E). In Ac_P10^prl-20^-infected cells, few P10 structures were detected and none were observed in a peri-nuclear location (Fig 5E). The data support the hypothesis that reduction of P10 levels (Fig 5A and 5B) is concomitant with reduction in nuclear lysis (Fig 5C and 5D) and the confocal analysis (Fig 5E) suggests that impaired development of P10 structures may provide the mechanistic link.

### Nuclear P10 has a role in occlusion body calyx formation

**Fig 6:**
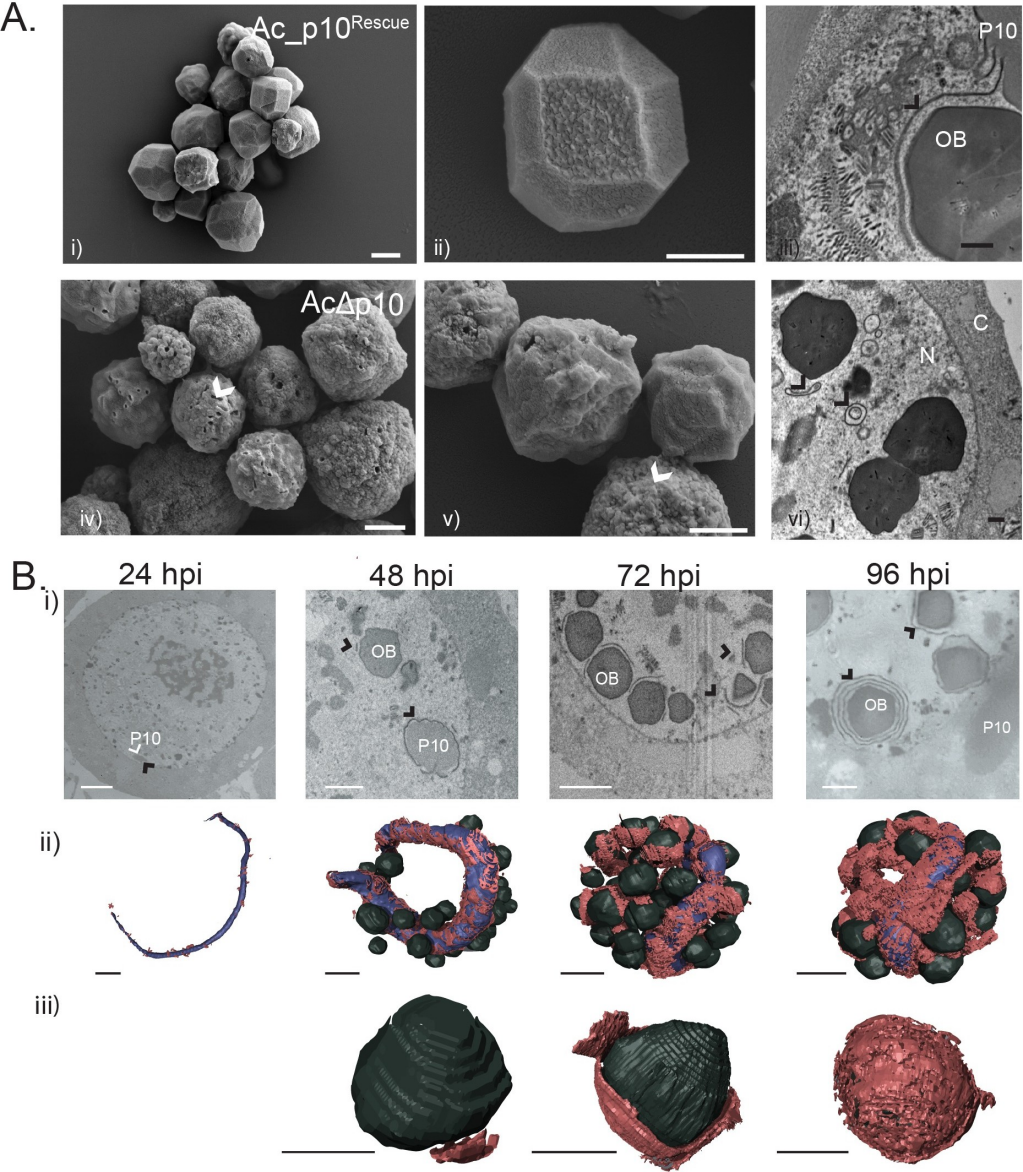
SEM and TEM images, and SBF-SEM 3D models, visualise the association between electron dense spacers, P10 and occlusion bodies in AcMNPV-infected TN-368 cells at 24, 48, 72 and 96 hpi. A) SEM (i, ii, iv, v) and TEM images (iii, vi) of occlusion bodies (OBs) from Ac_*p10*^Rescue^ and AcΔ*p10*-infected TN-368 cells. OBs from AcΔ*p10*-infected cells are pitted with a rough surface as indicated by arrow (iv, v) and are absent of a closely associated EDS as observed for Ac_*p10*^Rescue^ (vi). Scale Bar (i) 2 μm, (ii, iv, v) 1 μm (iii, vi) 500nm. B) SBF-SEM images (i) and surface generated models (ii, iii) from AcMNPV-infected TN-368 cells highlighting P10, EDS (black arrow) and OBs. Nuclear P10 (blue) is modelled as 24, 48, 72 and 96 hpi with EDS (red) and OBs (black). Detailed observations of OBs (iii) show the progressive encapsulation process of the OBs. Abbreviations: EDS electron dense spacers, OB occlusion bodies, C cytoplasm, N nucleus. Black arrows indicate EDS. Scale bar = 3 μm.

In addition to nuclear lysis or disintegration, it has also been suggested that P10 plays a role in calyx or PE formation, the final stage in the maturation of OBs [15, 20, 22]; although no one has hitherto been able to describe how P10 may be involved in such diverse functions. High-resolution SEM images of OBs isolated from Ac_*p10*^Rescue^ (Fig 6Ai and 6Aii) and AcΔ*p10-*infected cells (Fig 6Aiv and 6Av) showed that there were readily distinguishable morphological differences in OBs in the presence (Ac_*p10*^rescue^) or absence (AcΔ*p10*) of P10. The OBs extracted from Ac_*p10*^Rescue^-infected TN-368 cells predominantly presented a characteristic polyhedron shape with a smooth calyx; a few mis-formed or immature OBs were also detected (Fig 6Ai and 6Aii). Thust OBs extracted from AcΔ*p10-*infected cells exhibited a spherical phenotype that lacked the smooth outer layer (calyx/PE) and often resulted in OBs with a pitted surface (Fig 6Aiv and 6Av). This pitted surface is characteristic of an incomplete calyx/PE and subsequent loss of occlusion derived viruses (ODVs) during preparation of OBs; indicating a lack of ODV stability within OBs in the absence of a complete polyhedron envelope [38]. Measurements of OB diameter (data in S1 Fig), confirmed by a one-way ANOVA (P<0.05), showed a statistically significant difference (P<0.0001, n = 3) between OBs extracted from AcΔ*p10-*infected cells (mean = 2.521 μm) and either AcMNPV (mean = 2.896 μm) or Ac_*p10*^Rescue^ (mean = 2.915 μm). This gives a 12.7% and 13.6% reduction in the diameter of the OBs extracted from AcΔ*p10-*infected cells when compared to AcMNPV or Ac_*p10*^Rescue^ respectively. A Tukey’s multiple comparisons *post-hoc* analysis confirmed the mean size of the AcΔ*p10* OBs to be statistically different to both the AcMNPV (P = <0.0001) and Ac_*p10*^Rescue^ (P = <0.0001). The OBs derived from AcMNPV and Ac_*p10*^Rescue^ showed no statistical variation (P = 0.9569) in mean OB size.

We found from TEM images of Ac_*p10*^Rescue^- and AcΔ*p10-*infected cells that in the absence of P10, EDS failed to wrap around the periphery of OBs (Fig 6Aiii and 6Avi). Instead, EDS were observed to wrap onto themselves, forming self-aggregated spherical structures. This suggests that P10 may play a chaperone-like role in transferring the EDS to the surface of maturing OBs and supports the theory that P10 has a role in the formation of the calyx/PE. Furthermore, Fig 6 Aiii and 6Avi, demonstrated that EDS were present in the absence of P10, confirming that P10 is not a pre-requisite for EDS formation.

### Electron opaque spacers have an intimate association with nuclear P10 and occlusion bodies

To analyse the extent of the association between nuclear P10, EDS and OBs, we reconstructed 3D models of EDS (red), nuclear P10 (blue) and OBs (black) at different time-points after infection (Fig 6B). The presence of EDS were first observed at 24 hpi, identified only as small patches associated with the thin nuclear P10 fibrous body (Fig 6Bi and 6Bii). The OBs were absent or immature at 24 hpi (Fig 6Bi). By 48 hpi, extensive areas of nuclear P10 were coated with EDS, with some OBs also showing early signs of association with EDS (Fig 6Bi–6Biii). At 72 hpi, large areas of P10 nuclear structure were thickly coated with EDS with some appearing to form aggregates that branch off the main P10-associated EDS (Fig 6Bii). In multiple cells observed at 72 hpi, EDS were also aggregated in a layer covering large regions of the OB surface (Fig 6Biii), suggestive of OBs being encapsulated by the EDS. By 96 hpi, the majority of the nuclear P10 and OB surfaces were coated with at least one layer of EDS (Fig 6Bii and 6Biii). Interestingly, the 3D models of AcMNPV-infected cells indicated a continued production of EDS through-out virus infection resulting in multiple layers of EDS around a single OB at 96 hpi. At this time, we estimated that 80% of OBs were associated with EDS and approximately 50% of OBs were completely encapsulated within a layer of EDS.

These models highlight the intimate relationship between P10, EDS and OB structures and provide convincing evidence of a chaperone-like role for P10 in the formation of the polyhedral envelope or calyx.

## Discussion

In this study we used high-resolution 3D modelling of AcMNPV-infected insect cells to determine the size, shape, spatial and temporal association of key virus structures over the course of infection, which has enabled us to propose a mechanism for the possible role of P10 in nuclear lysis and OB maturation.

SBFSEM enabled large volumes of high-resolution data to be captured to generate detailed 3D-images of AcMNPV ultrastructure, at a resolution comparable to previously documented TEM images [5, 23, 39]. To generate similar large volumes of data would be difficult and time consuming using alternative microscopy methods.

The 3D modelling of whole insect cells has provided new details on the independent formation of two distinct P10 structures that form during baculovirus infection. These were identified as a dynamic cytoplasmic perinuclear structure and a nuclear worm-like structure (Fig 2A). The characterisation of these structures at different times after infection has revealed the temporal changes involved in the formation of both P10 structures, which provide evidence for the role of P10 in the mechanisms that effect nuclear lysis and OB maturation in cultured cells.

### Cytoplasmic P10 structures reveal a mechanism for nuclear lysis to release occlusion bodies

Previous studies using anti-tubulin and P10-specific antibodies showed that P10 structures associate with microtubules and could have a role in the reorganisation of the cytoskeleton as well as the initiation of P10 structure formation [30, 31]. Depolymerisation of the microtubule network with colchicine prevented formation of P10 filaments and a yeast two-hybrid experiment identified an interaction between P10 with host-cell tubulin [31]. However, it was noted that the thicker P10 tubules that form later, and which merge to form a perinuclear cage-like structure, are not closely associated with microtubules [30, 40]. Similar P10 filamentous structures have been observed with entomopoxvirus formed by the filament-associated late protein of entomopoxviruses (FALPE) [9]. FALPE shares structural and functional similarities to baculovirus P10 with a close association to the entomopoxvirus occlusion bodies [41].

**Fig 7:**
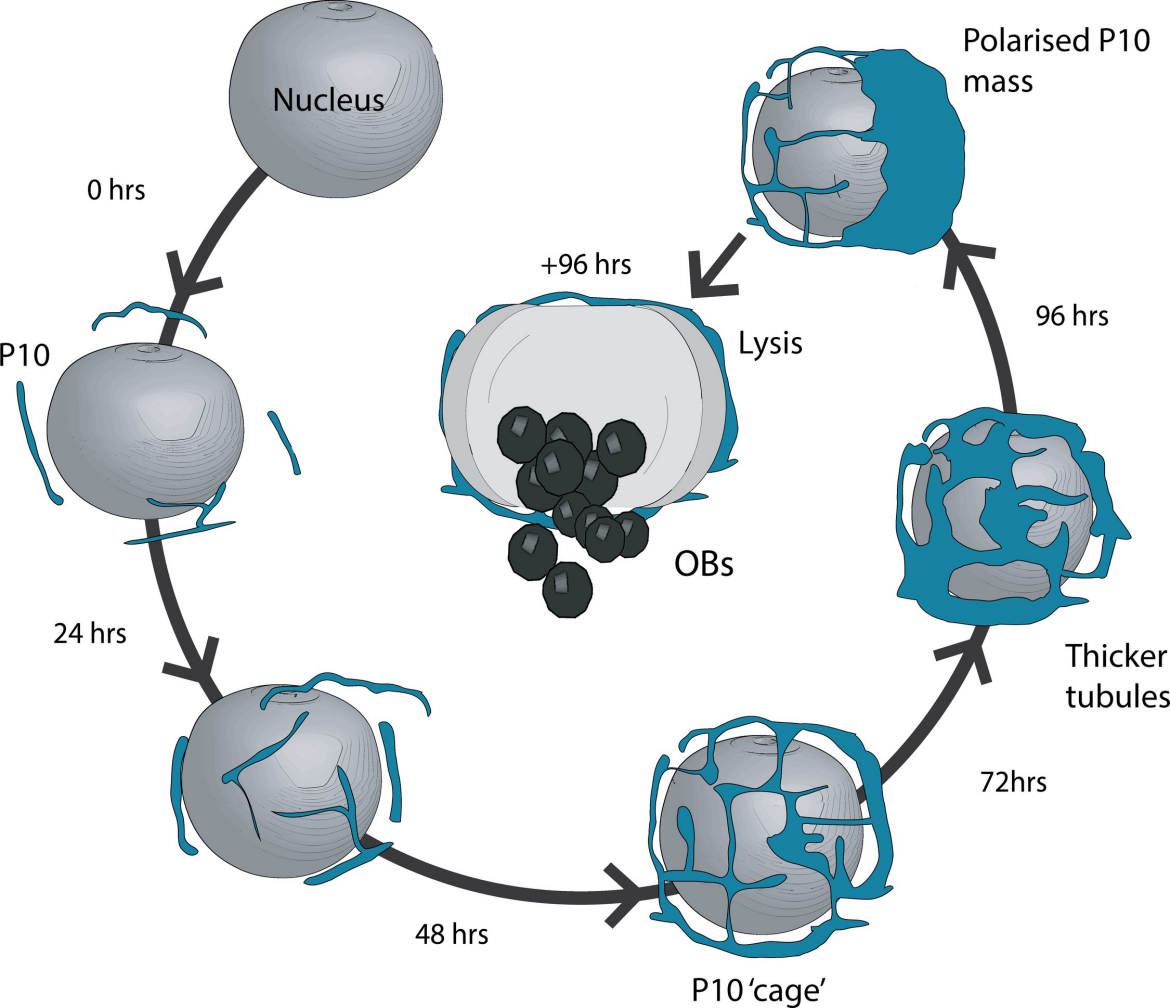
Structural model for the role of cytoplasmic P10 in nuclear lysis. P10 (blue) presents as a thin filamentous structure that develop into a complex network of P10 tubules that wrap around the nucleus forming a peri-nuclear cage-like structure. The cage-like structure continue to remodel over time and by 96 hpi most P10 has coalesced into a polarised mass. This final remodelling is associated with loss of nuclear integrity, resulting in nuclear lysis and release of occlusion bodies.

The SBFSEM images generated in this study enabled us to create detailed 3D models of cytoplasmic P10, which confirm the formation of the perinuclear cage structure proposed in the earlier confocal microscopy data. Furthermore in this study, we show that the perinuclear cage structure is dynamic and re-models over time, eventually coalescing into a polarised mass of P10 that is concomitant with the time of cell lysis (around 96 hpi in cell culture). From these data we can propose a model (Fig 7) in which cytoplasmic P10 first assembles into filaments using a scaffold of microtubules. These later (48 to 72 hpi) develop into thicker, longer tubules that interact to form a perinuclear network or cage-like structure that may stabilise the nucleus allowing time for OBs to develop and mature.

Finally at around 96 hpi, as the cage-like structure continues to remodel, P10 withdraws from the tubules coalescing into a polarised mass. At some stage in this final process, our model predicts that the integrity of the nuclear membrane is destabilised causing nuclear lysis and release of OBs, which in nature would assist in OB dispersal for horizontal transmission after other viral factors have liquefied the host (Fig 7). Our model is supported by the timing of this event that coincides with nuclear lysis of AcMNPV-infected TN-368 cells as observed by light microscopy [15]. Our model is also consistent with observations made during these studies (Fig 5D), and historically, that deletion of *p10* results in both the absence of P10 structures and the abrogation of nuclear lysis [15, 17]. One earlier study examining the role of structural domains of the P10 protein through the construction of deletion mutants, concluded that the process of nuclear lysis continued in the absence of an identifiable P10 structure [14] and this conflicts with our proposed model, in which cell lysis is dependent on the formation of P10 structures. However, more recent studies have presented TEM and confocal images of cells infected with viruses containing similar deletions to those described in [14]; these data clearly showed that P10 structures were formed in these cells [42, 43], which is consistent with our model.

### Nuclear P10 has a role in polyhedral envelope or calyx formation

Early reports on P10 proposed a role in OB maturation, which was a result of TEM studies on baculovirus structures [5, 23] that observed P10 in close association with EDS and OBs [19, 22, 29, 44, 45]. These OBs were observed to be surrounded by EDS and it was suggested that EDS aided formation of the PE/calyx by a process of condensing and compression [39]. Even though a number of studies have commented on an association between P10, EDS and the PE/calyx [19, 22, 29, 45], there was little evidence for a mechanism to explain the role of P10 in calyx formation, with at least one study sceptical of the involvement of P10 [17].

Using high-resolution SEM images of OBs, this study provides further evidence that P10 is required to form an intact PE/calyx and 3D modelling of P10 provides new evidence for a close spatial and temporal relationship between P10, EDS and OBs. Using the acquired data, we show an increase in formation and association of EDS with both P10 and OBs during the later phase of infection (48–72 hpi). Nuclear P10 becomes almost entirely coated with EDS that appear to stack up upon each other and by 96 hpi, multiple OBs are fully encapsulated by EDS. Our data supports the suggestion [35] that P10 plays a chaperone-like role in transferring the EDS to the surface of maturing OBs as clearly depicted in the images in Fig 6B.

It is important to note that *p10* deletion mutants show no impairment in the production of polyhedral envelope protein (pp34) or EDS production [19] but fail to form an intact calyx [22] as confirmed here by SEM (Fig 6Ai). In the absence of P10, we also noted that the EDS failed to wrap around the periphery of OBs and instead formed self-aggregated spherical structures (Fig 6Aiv). This confirms a requirement for P10 in the formation of the PE. It is also possible that the principle function of P10 is to maintain nuclear integrity via the formation of its cage structure to enable the maturation of the occlusion body with an intact calyx. A process that provides protection from environmental hazards and limits loss of ODV.

### Hyper-expression of P10 is required for nuclear lysis

This study reports the first instance of targeted control of *p10* expression through modification of its promoter, and provides evidence that hyper-expression of *p10* is required for P10 to fulfil its biological role in mediating nuclear lysis. In our study, we detected a direct correlation between the amount of P10 protein synthesised and the ability of cells to undergo nuclear lysis, with a critical amount of P10 required to maximise nuclear lysis and hence aid OB dispersal. Previous studies examining the *p10* promoter also observed a reduction in expression with progressive deletions made to the 5’ non-coding region of the promoter [46, 47]. However, in these studies the *p10* coding region had been replaced with that of the CAT reporter gene so the effect on the levels of P10 protein was not recorded. Tni Hi5 cells infected with Ac_P10^prl-12^ gave comparable levels of P10 protein to that detected in both AcMNPV and Ac_P10^Rescue^, and we observed formation of normal P10 structures and full nuclear lysis. We have no explanation for why a promoter deletion should result in wild type levels of protein production, but its sequence was confirmed in the recombinant virus. In contrast, the other promoter deletions resulted in low levels of P10, few or no recognisable P10 structures and low levels of nuclear lysis. The relatively high levels of *p10* expression observed in Ac_P10^prl-12^ were not previously documented [46, 47]. We can derive from this study that manipulation of the *p10* promoter decreased P10 protein levels that resulted in a diminished P10 cage structure with a concomitant decrease in nuclear lysis and abrogation of OB release into the culture medium.

It has long been debated why both *p10* and *polh* are under control of the strong, very late promoters that are hyper-expressed in baculovirus-infected cells. Large quantities of polyhedrin are required to form the paracrystalline array that comprises the structure of OBs into which ODV are embedded and protected [48, 49]. P10, which is also produced in large quantities, is non-essential for the production of orally infective ODV *in vivo* [12, 15]. However, that there has been a strong evolutionary pressure to preserve the characteristic P10 structure indicates an advantage for *p10* [50, 51]. One suggestion is that expression of *p10* promotes the dispersal and spread of the viral progeny [50]. Our data suggest that large quantities of P10 are required to form the cytoplasmic cage-like structure that provides a mechanistic action to mediate nuclear lysis and OB release.

In conclusion, the 3D modelling of AcMNPV structures from a high-resolution data set identified the formation of two independent P10 structures in cultured cells. Cytoplasmic P10 forms a dynamic perinuclear cage-like structure that we propose plays a crucial role in mediating nuclear lysis and OB dispersal. Hyper-expression of *p10* is required for cage formation and consequently nuclear lysis. The nuclear form of P10 comprises a single vermiform structure that most likely plays a chaperone-like role in facilitating the maturation of OB by promoting the transfer of EDS to form the polyhedral PE/calyx.

## Materials and methods

### Cells and viruses

Cells used were derived from Tn Hi5 (Invitrogen) and TN-368 [36] and were maintained at 28°C in ESF921 (Expression Systems) or TC100 media (Invitrogen) supplemented with 10% foetal bovine serum, respectively. AcMNPV clone 6 virus was propagated and amplified using standard methods [52]. The construction of a p10-knockout virus, AcΔp10, rescue virus, Ac_p10^rescue^ (S2 Text), and series of promoter deletions in the 5’ untranslated sequence of *p10* mRNA (S3 Text) were produced by co-transfecting Sf9 cells with *Bsu*361-digested AcΔ*p10*_*lacZ* DNA and designated transfer vector (S1 Text), followed by plaque-purification and amplification of viruses [52]. Oligonucleotide sequences are provided in S2 Table.

### Virus infections and preparation for microscopy

AcMNPV-infected TN-368 cell pellets at 0, 24, 48, 72 and 96 hpi were washed with PBS and placed into primary fixative (2% paraformaldehyde, 2.5% glutaraldehyde and 0.1% tannic acid in 0.1M sodium cacodylate buffer, pH 7.4). Six 5-second microwave bursts at 5 second intervals (Russel Hobbs, Digital microwave, 800W) were applied to the cell pellets and fixative solution for rapid microwave fixation [53], followed by a 15 minute incubation at room temperature. The cell pellet was washed four times by re-suspending in 0.1M sodium cacodylate buffer (pH 7.4) at room temperature for 5 minutes followed by low speed centrifugation. Secondary fixation was performed with 1% osmium tetroxide in 0.1 M sodium cacodylate buffer, followed by six 5-second microwave blasts and a 1 hour incubation at room temperature. The cell pellet was rinsed x5 with distilled water, centrifuged to prevent sample loss, and then 2% osmium tetroxide in 0.1 M sodium cacodylate buffer was applied and incubated for 40 minutes at room temperature. After washing in water again, samples were dehydrated in an ascending ethanol series to a final concentration of 100%, with an intermediate incubation overnight at 4°C in 70% ethanol containing 2% uranyl acetate. After dehydration, the cell pellet was infiltrated and embedded in Epon 812 resin and polymerised at 70°C for 18 hours.

### Serial block face scanning electron microscopy

The resin embedded samples were sectioned and imaged using a Gatan 3View system including the 3View2XP stage and 3VBSED detector (Gatan, Abingdon, UK) in combination with a Zeiss Merlin compact VP SEM (Zeiss, Cambridge, UK). The sample was imaged using an accelerating voltage of 4 kV, aperture size of 30 μm and dwell time was 3 μs. Variable pressure was between 30–45 Pa. Pixel size was 6.9–15 nm for 500–700 sections at 100nm each. See S1 Table for imaging parameters for each time point.

### Image analysis

The raw image datasets were acquired using a digital micrograph format (dm4; Gatan, Abingdon, UK). The images were then converted to obtain stacked image files (mrc) for data processing using a free iMOD [54] and AMIRA (ThermoFisher) software packages. Structures were selected in segmentation, using both automatic (thresholding and interpolation) and manual tools to define viral structures. Structures were modelled based on their classic morphological features. The 3D volume rendered images were used for precise measurements. P10 diameter data were taken at 10 representative sites for cytoplasmic P10 fibrils/tubules and 10 representative sites for nuclear P10 per cell. The data were analysed using GraphPad Prism 7 (GraphPad Software Inc., USA).

### Preparation and imaging of occlusion bodies

SEM was performed on OBs extracted from baculovirus-infected TN-368 cells (at 5 MOI) at 7 dpi as previously described [45]. Purified OBs were fixed in 4% (v/v) formaldehyde in PIPES buffer for 1 hour, washed once in PIPES buffer and then dehydrated in an ascending ethanol series to 100%, for 10 minutes each. The dehydrated OBs were densely seeded onto glass coverslips and sputter-coated with gold (Automatic sputter coater, Agar Scientific, Stansted, UK). The samples were imaged using a Zeiss Merlin Compact VP SEM using an accelerating voltage of 4kV. Post-acquisition image processing was performed with Image J and analysed using GraphPad Prism 7.

### SDS-PAGE and western blot analysis

Proteins were separated in 15% gels using the Mini-PROTEAN system (Bio-Rad) as previously described [28] and stained with Coomassie blue or immuno-stained using rabbit anti-P10 (CFELDSDARRGKRSSK, 1:500; Genscript) antisera with alkaline phosphatase conjugated secondary antibody (1:30,000; Sigma-Aldrich). Colorimetric images were taken using a ChemiDoc MP imaging system (Bio-Rad).

### Immunofluorescence staining

Immunofluorescence staining of infected TN-368 cells using rabbit anti-P10 antibody (1:700) and Alexa-fluor 488 -conjugated anti-rabbit secondary antibody (1:1000) was as described previously [30, 55].

### Confocal microscopy and image processing

For image acquisition, a Zeiss LSM 510 meta laser scanning microscope using the 488nm excitation line of the argon laser was used. Fluorescence was detected using a 488/543 nm dichroic beam splitter and a 505-530nm band pass filter for Alexa-Fluor 488. Images were acquired using an oil immersion objective; Plan-Apchromat 63X (1.4 numerical aperture). Post-acquisition image processing and Z-stack image projections were processed using LSM 5 image Browser (Zeiss, Cambridge, UK).

### Statistical analysis

Data were analysed using software package GraphPad Prism 7. Statistical analysis was performed using both one- and two-way ANOVA with a Tukey’s multiple *post-hoc* test. A p-value of <0.05 was considered statistically significant.

## Supporting information

S1 Text Construction of intermediate virus that replaces *p10* coding region with *lac*Z. (DOCX)

S2 Text *p10*-deletion (pAcΔ*p10*) and *p10*-rescue (pAc_*p10*^Rescue^) plasmids. (DOCX)

S3 Text Transfer vectors containing *p10* promoter deletions. (DOCX)

S1 Table Imaging parameters for acquisition of data by SBF-SEM. (TIF)

S2 Table Oligonucleotide primer sequences used for virus constructs. (TIF)

S1 Fig Comparisons of SEM-imaged occlusion bodies. Box and whiskers plot of mean occlusion body (OB) diameter (nm) from AcMNPV, Ac_p10Rescue and AcΔp10-infected TN-368 cells; n = 100. Bars represent 5–95% range with outliers plotted. ANOVA (P<0.05) represents statistical difference between cohort, **** = < 0.0001 or ns (no significance difference). Images acquired using a SEM Hitachi S-3400 at Accelerating voltage of 5 Kv.
(TIF)

S1 Video Video showing a collection of stacked slices obtained from AcMNPV-infected TN-368 cells using SBF-SEM at 96 hpi. (MP4)

S2 Video Video showing the 3D reconstruction of AcMNPV-infected TN-368 cell at 24 hpi. (MPG)

S3 Video Video showing the 3D reconstruction of AcMNPV-infected TN-368 cell at 48 hpi. (MPG)

S4 Video Video showing the 3D reconstruction of AcMNPV-infected TN-368 cell at 72 hpi. (MP4)

S5 Video Video showing the 3D reconstruction of AcMNPV-infected TN-368 cell at 96 hpi. (MPG)
