# Supplementary material for: In cultured cells the baculovirus P10 protein forms two independent intracellular structures that play separate roles in occlusion body maturation and their release by nuclear disintegration
Source: PLoS Pathog. 2019 Jun 10;15(6):e1007827. doi: 10.1371/journal.ppat.1007827 (PMC6557513; doi:10.1371/journal.ppat.1007827)
Supplement: S1 Text — (DOCX) [file ppat.1007827.s001.docx]

**S1 Text: Construction of intermediate virus that replaces p10 region with LacZ**

To generate the plasmids used in this study, an intermediate virus that replaces *p10* with *lacz* was first constructed. To generate pAc∆*p10*_*lacZ,* pAcUW1 was digested with restriction endonucleases *Bgl*ll and *Bcl*l to remove the partial *p10* sequence [1] . This was then replaced with *lacZ* excised from pCH110 by digestion with *Bgl*ll and *Bam*Hl. To generate a homogeneous stock of recombinant virus, insect Sf9 cells were co-transfected with wild-type AcMNPV C6 DNA [2] and pAc∆*p10*_*lacZ* plasmid and subjected to plaque purification to make Ac∆*p10*_*lacZ* virus [3].
