# Supplementary material for: In cultured cells the baculovirus P10 protein forms two independent intracellular structures that play separate roles in occlusion body maturation and their release by nuclear disintegration
Source: PLoS Pathog. 2019 Jun 10;15(6):e1007827. doi: 10.1371/journal.ppat.1007827 (PMC6557513; doi:10.1371/journal.ppat.1007827)
Supplement: S2 Text — (DOCX) [file ppat.1007827.s002.docx]

**S2 Text: P10-deletion (pAc∆*p10*) and P10-rescue (pAc_*p10*^Rescue^) plasmids**

To remove the partial *p10* sequence, pAcUW1 was digested with *Bgl*ll and *Bcl*l to excise *p10* and re-ligated to produce pAc∆*p10*. pAc_*p10*^Rescue^ was constructed by the initial removal of partial *p10* sequence using *Pac*l and *Bcl*l sites of pAcUW1. This was replaced with full length *p10*, PCR amplified from AcMNPV DNA using pAcLG1 and pAcLG4 (S2 Table). These primers introduced *Pac*l and *Bcl*l sites at the 5’ and 3’ ends for insertion to digested pAcUW1.
