# Supplementary material for: In cultured cells the baculovirus P10 protein forms two independent intracellular structures that play separate roles in occlusion body maturation and their release by nuclear disintegration
Source: PLoS Pathog. 2019 Jun 10;15(6):e1007827. doi: 10.1371/journal.ppat.1007827 (PMC6557513; doi:10.1371/journal.ppat.1007827)
Supplement: S3 Text — (DOCX) [file ppat.1007827.s003.docx]

**S3 Text: *p10* transfer vectors containing promoter deletions**

To generate Ac_P10^prl-4^, Ac_P10^prl-8^, Ac_P10^prl-16^, Ac_P10^prl-20^ a series of deletions were made in the DNA encoding the 5’ untranslated sequence of *p10* mRNA (S1 Figure). These were constructed using a two-step PCR. An oligonucleotide (PrimeLG_ECOR1_F, S1 Table) binding upstream of the *p10* transcription start site was paired with one of a number of primers (S2 Table) that bound at various points upstream of the *p10* ATG translation initiation codon (S1 Figure). These PCR fragments were then used as templates in a second round of reactions using PrimeLG_ECOR1_F and a series of unique primers that added the first 18 bp of the *p10* coding region to the product (S1 Figure). The PCR amplicons were digested with *Sph*1 and *Acl*1 and cloned into to pAc_*p10*^Rescue^ digested with complementary restriction endonucleases.
