# Supplementary figures and images for: In cultured cells the baculovirus P10 protein forms two independent intracellular structures that play separate roles in occlusion body maturation and their release by nuclear disintegration
Source: PLoS Pathog. 2019 Jun 10;15(6):e1007827. doi: 10.1371/journal.ppat.1007827 (PMC6557513; doi:10.1371/journal.ppat.1007827)

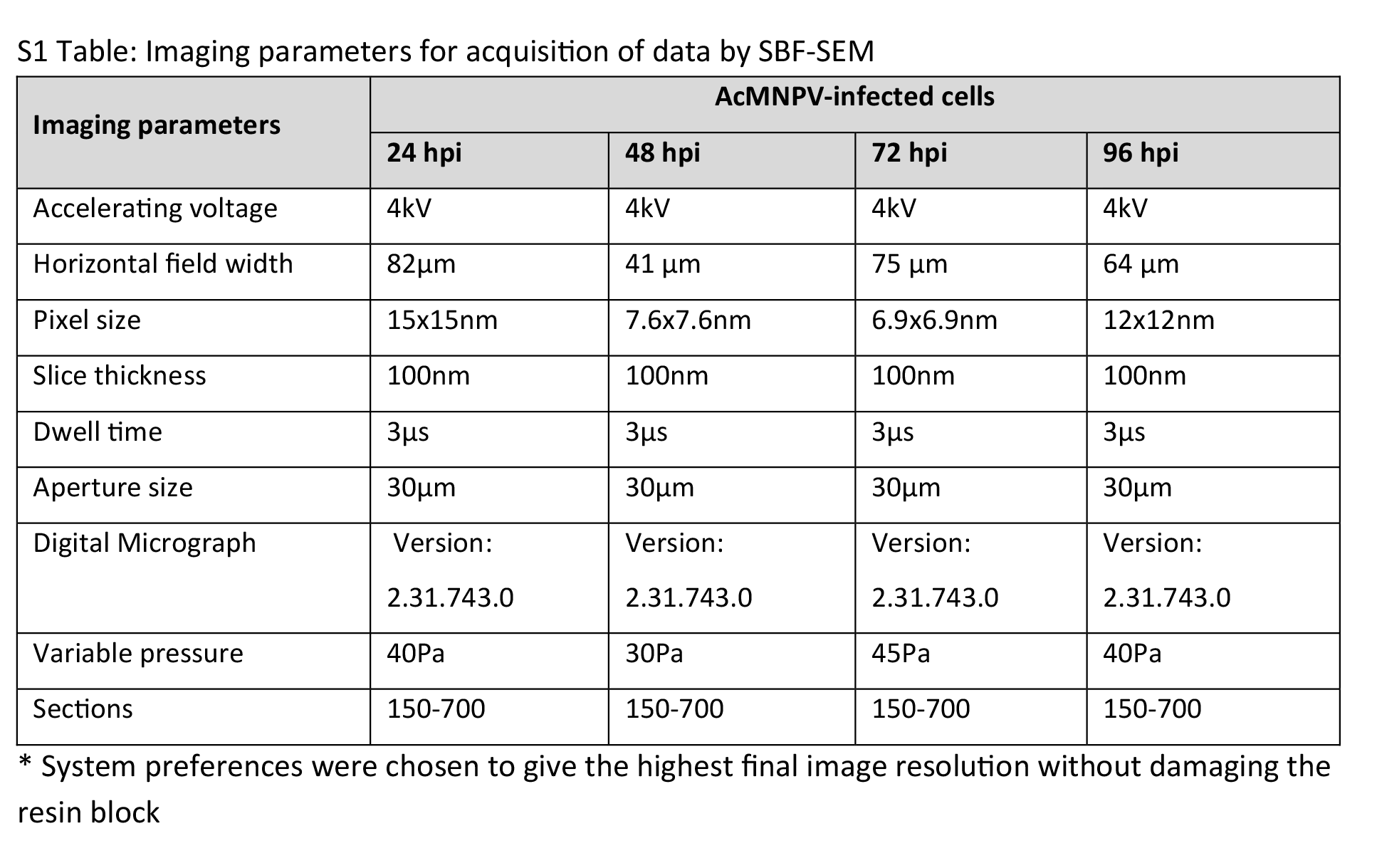

Supplement: S1 Table — (TIF) [file ppat.1007827.s004.tif]

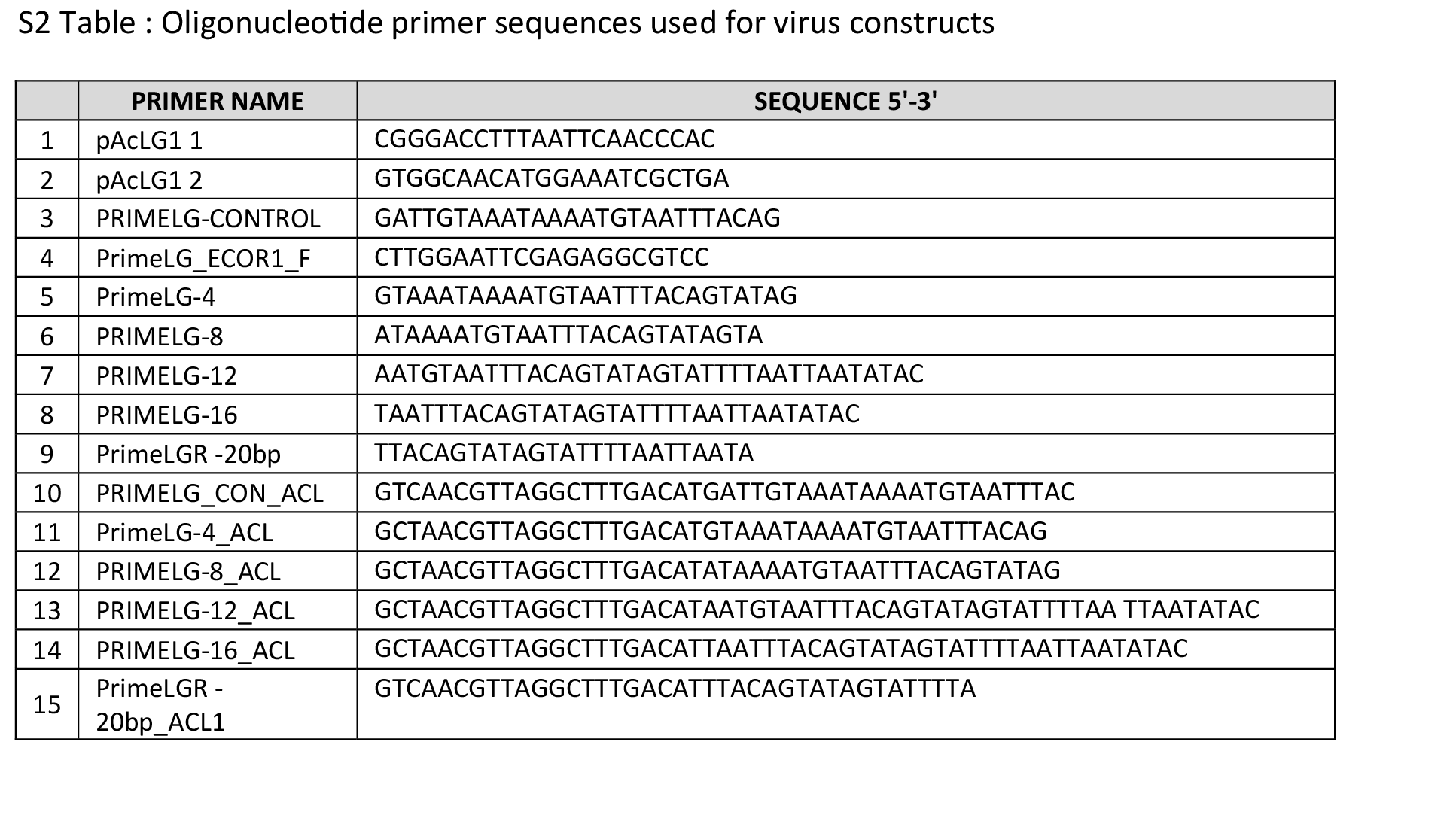

Supplement: S2 Table — (TIF) [file ppat.1007827.s005.tif]

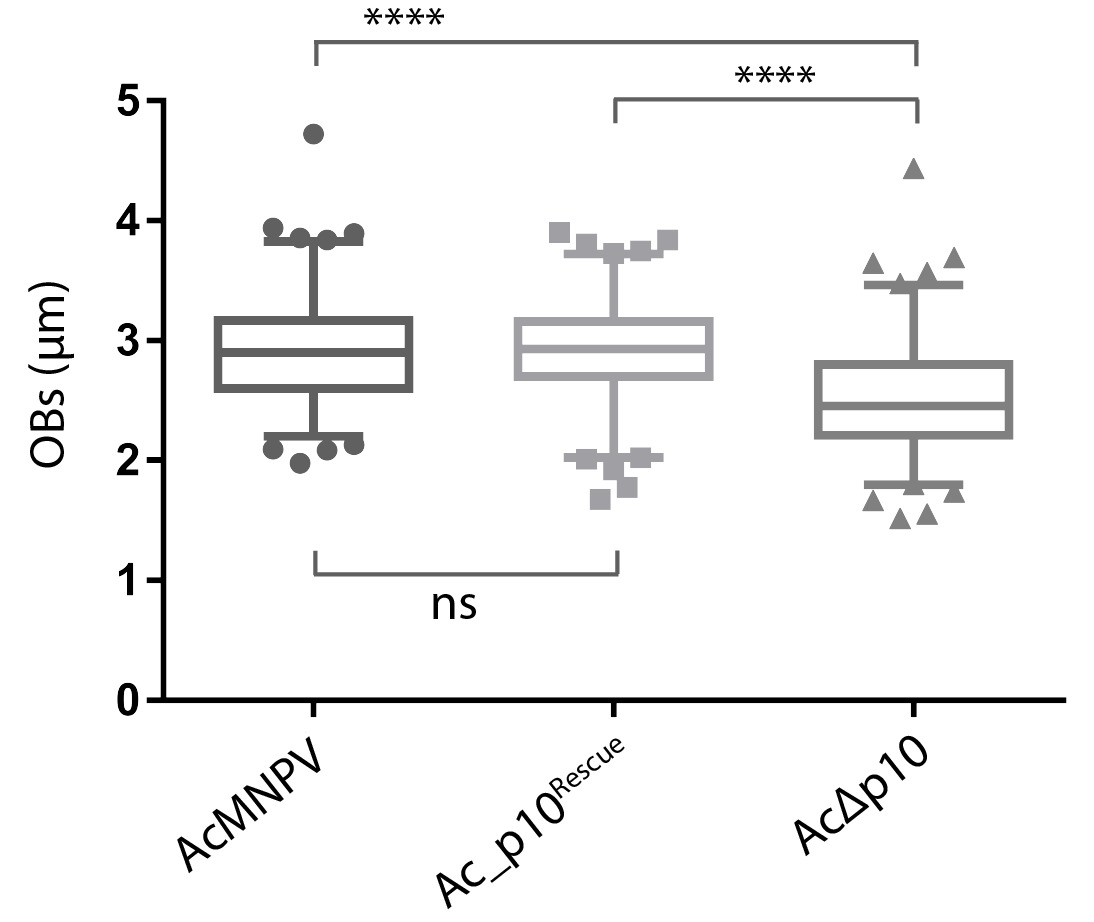

Supplement: S1 Fig — Box and whiskers plot of mean occlusion body (OB) diameter (nm) from AcMNPV, Ac_p10Rescue and AcΔp10-infected TN-368 cells; n = 100. Bars represent 5–95% range with outliers plotted. ANOVA (P<0.05) represents statistical difference between cohort, **** = < 0.0001 or ns (no significance difference). Images acquired using a SEM Hitachi S-3400 at Accelerating voltage of 5 Kv. (TIF) [file ppat.1007827.s006.tif]
